# The nitrogen-vacancy defect in Si_1-x_Ge_x_

**DOI:** 10.1038/s41598-025-94959-2

**Published:** 2025-03-26

**Authors:** Stavros-Richard. G. Christopoulos, Navaratnarajah Kuganathan, Efstratia Sgourou, Charalampos Londos, Alexander Chroneos

**Affiliations:** 1 Department of Computer Science, School of Computing and Engineering, University of Huddersfield, Huddersfield, HD4 6DJ UK; 2 Centre for Computational Science and Mathematical Modelling, Coventry University, Coventry, CV1 2TU UK; 3 Department of Materials, Imperial College London, London, SW7 2AZ UK; 4 Physics Department, Solid State Physics Section, National and Kapodistrian University of Athens, Athens, 157 84 Panepistimiopolis Zografos Greece; 5 Department of Electrical and Computer Engineering, University of Thessaly, Volos, 38221 Greece

**Keywords:** Si_1 − x_Ge_x_, Defects, DFT, Doping, Binding energy, Materials science, Condensed-matter physics, Materials for devices

## Abstract

Defect processes and energetics in semiconducting alloys is scientifically and technologically important as silicon germanium (Si_1 − x_Ge_x_) is a mainstream nanoelectronic material. It is established that point defects and defect clusters have an increasing role in the physical properties of Si_1 − x_Ge_x_ particularly with the ever-decreasing critical dimensions of nanoelectronic devices. Nitrogen-vacancy defects in Si_1 − x_Ge_x_ are bound and have the potential to change the optical and electronic properties and thus need to be investigated as absolute control is required in nanoelectronic devices. The nitrogen-vacancy defects are not extensively studied in Si_1 − x_Ge_x_ random semiconductor alloys. Here we employ density functional theory (DFT) in conjunction with the special quasirandom structures (SQS) method to calculate the binding energies of substitutional nitrogen-vacancy pairs (N*V*) in Si_1 − *x*_Ge_*x*_ alloys. This is a non-trivial problem as the energetics of these defect pairs are dependent upon the nearest neighbour Ge concentration and the composition of Si_1 − *x*_Ge_*x*_. The criterion for N*V* stability is binding energy and here it is shown that the most bound N*V* defects will form in high Si-content Si_1 − *x*_Ge_*x*_ alloys.

## Introduction

Si_1 − x_Ge_x_ is applicable in electronic applications because of its higher carrier mobility, and wider range of band gap as compared to silicon (Si)^1–10^. The dominance of Si for microelectronic devices over other materials such as Si_1 − x_Ge_x_ or germanium (Ge) was established mainly due to its native oxide (SiO_2_) that was resilient and appropriate properties for scaling purposes^5–8^. In the past two decades the ability to use high dielectric constant (high-*k*) materials in device level has unveiled the possibility to implement higher carrier mobility (i.e. higher conductivity) materials including Si_1 − x_Ge_x_ and Ge^9–13^.

Nitrogen (N) is a Group V element such as phosphorous (P) and arsenic (As), however, it is not an appropriate n-type dopant in Si, Ge or Si_1 − x_Ge_x_. At any rate the introduction of N in Si or Ge is important and may lead to dislocation locking that enhances the mechanical properties of wafers^14^. The mechanical properties are significant for ultra-large-scale integration technologies as Si wafers need to withstand demanding processing steps without breaking^14^. It is established that nitrogen in Si can decrease the microdefect (A-swirls or D-defects) and void content during float-zone crystal growth^15,16^. In germanium, nitrogen can impact the n-type dopants defect processes and the passivation of the material^17,18^. The N*V* defects in group IV semiconductors can become increasingly important in bio-imaging, sensors, and nanoscale thermometry^19–23^.

There are previous theoretical modelling studies that have investigated the interaction of nitrogen with vacancies in Si and Ge, however, there are only very few analogous studies of these defects in Si_1 − x_Ge_x_. As Si_1 − x_Ge_x_ is a mainstream nanoelectronic material its nitrogen defect processes are not only of scientific but also of technological importance. In the present study DFT calculations are employed to trace the energetically favourable vacancy-nitrogen configurations in Si_1 − x_Ge_x_ and to study their nearest neighbour environment and its impact on the defect energetics.

## Computational methods

In the present study, we examined the binding nature of nitrogen with vacancy defects in Si_1 − x_Ge_x_ using the plane wave DFT code CASTEP^24,25^. The correlation and the exchange interactions are described using the corrected density functional of Perdew, Burke, and Ernzerhof (PBE)^26^, the generalized gradient approximation (GGA), BFGS (Broyden-Fletcher-Goldfarb-Shanno) geometry optimisation algorithm in conjunction with the ultrasoft pseudopotensials^27^.

For the calculations we used a 64-atomic site supercell constructed by two 32-atoms SQS cells that were derived previously^28^. The plane wave basis was set by choosing the level of convergence of the atomic energies to 0.000544 eV/atom as this ensures a very high level of precision in the optimization process, a 2 × 2 × 2 Monkhorst-Pack (MP)^29^ k-point grid. We performed seven sets of calculations for different SQS configurations for the following concentrations of Si_1 − x_Ge_x_ (x = 0.125, 0.25, 0.375, 0.5, 0.625, 0.75, 0.875).

For each of these seven sets we performed 128 calculations for all the unique different nitrogen-vacancy pairs, 32 calculations for all the unique different nitrogen sites, 32 calculations for all the unique different vacancy sites and one calculation for the initial structure. Thus, we performed 1351 calculations in total. We employed the Defects and Impurities Setup (DIMS) tool^30^ to streamline the process and avoid potential errors associated with the manual setup of the calculations. The visualizations presented in this paper were generated utilizing the Visualization for Electronic and Structural Analysis (VESTA) software (version 3)^31^.

The binding energy of a NV defect in Si_1 − x_Ge_x_ was calculated using:

1 $$\:{\text{E}}_{\text{b}\text{i}\text{n}\text{d}\text{i}\text{n}\text{g}}\left(\text{N}\text{V}\right)=\text{E}[\text{N}\text{V}{]}_{\text{s}\text{u}\text{p}\text{e}\text{r}\text{c}\text{e}\text{l}\text{l}}+\text{E}[\text{S}\text{i}\text{G}\text{e}{]}_{\text{s}\text{u}\text{p}\text{e}\text{r}\text{c}\text{e}\text{l}\text{l}}-\text{E}[\text{N}{]}_{\text{s}\text{u}\text{p}\text{e}\text{r}\text{c}\text{e}\text{l}\text{l}}-\text{E}[\text{V}{]}_{\text{s}\text{u}\text{p}\text{e}\text{r}\text{c}\text{e}\text{l}\text{l}}\:$$

where $\:\text{E}[\text{N}{]}_{\text{s}\text{u}\text{p}\text{e}\text{r}\text{c}\text{e}\text{l}\text{l}}$ is the total energy of a single N atom substitutionally doped in the supercell of Si_1 − *x*_Ge_*x*_ and $\:\text{E}[\text{V}{]}_{\text{s}\text{u}\text{p}\text{e}\text{r}\text{c}\text{e}\text{l}\text{l}}$ is the total energy of a supercell containing a single vacancy.

The electronic structures and charges of the relaxed configurations were analysed using DFT simulations as implemented in the Vienna Ab initio Simulation Package (VASP)^32^. A plane wave basis set with a 500 eV cut-off and a 4 × 4 × 4 Monkhorst-Pack k-point mesh^29^, along with the GGA-PBE exchange-correlation functional^26^ were used. Bader charge analysis^33^ was used to elucidate the charge transfer and distribution among the atoms.

## Results and discussion

### Modelling silicon germanium

It is established that the DFT investigation of even the simplest defect clusters in random alloys and solid solutions is complicated because it is necessary to calculate all the possible configurations with respect to at least first nearest neighbour environments. This in essence requires a very extensive number of possibilities and hence calculations in large supercells. The SQS method is an efficient way to reproduce the vast local environments existing in solid solution and at the same time reduce the number of calculations and supercell size^34,35^. They have been previously used to study the defect-dopant properties of solid solutions including binary (for example Si_1 − *x*_Ge_*x*_, Sn_1 − *x*_Ge_*x*_) and ternary (for example Si_1 − *x*−*y*_Ge_*x*_Sn_y_) group IV random alloys^28,36–39^.

In a nutshell the SQS are designed small-unit-cell periodic structures that efficiently mimic the near neighbour pair and multisite correlation functions of the random substitutional alloys^34,35^. As they are atomistic models there is a distribution of local environments, which will exist in the real random alloys. For the system considered here the Si or Ge atoms can be surrounded by various Si_n_Ge_4−n_ coordination shells (where *n* = 0 to 4) and this is the basis for a distribution of local environments. These local environments will have an important impact on the dopant-defect interactions^38^. The efficacy of the SQS technique to model Si_1 − x_Ge_x_ (x = 0.125, 0.25, 0.375, 0.5, 0.625, 0.75, 0.875) has been discussed in previous studies^40,41^.

### N*V* defect

**Fig. 1:**
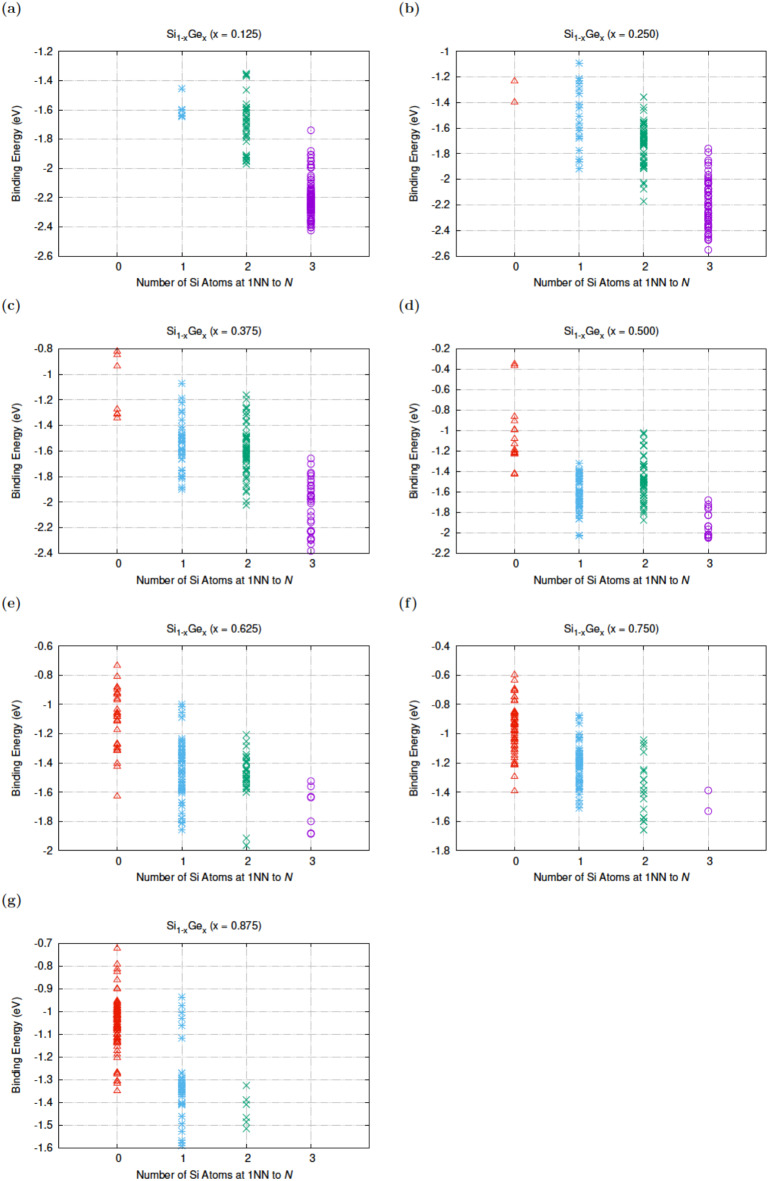
The binding energy of N*V* defects as a function of the number of Si first nearest neighbour atoms to the N atom in Si_1 − x_Ge_x_ (x = 0.125, 0.25, 0.375, 0.5, 0.625, 0.75, 0.875).

A schematic representation of the seven 32-atom SQS Si_1 − x_Ge_x_ cells is given in Fig. 1 of Ref. 28. We have considered here 64-atom supercells and calculated all the possible N*V* defects within these supercells. Figure 1 reports the impact of the first nearest neighbour Si or Ge lattice atoms to the N substitutional atom. It is predicted that all the nearest neighbour N*V* defects are bound and this is qualitatively different to the picture gained when considering *E*-centers and in particular phosphorous-vacancy pairs (P*V*) in Si_1 − *x*_Ge_*x*_^40^. In particular, DFT studies predicted that up to 62.5% Si content there were also positive P*V* binding energies^40^. This in turn meant that P*V* pairs in Si_1 − *x*_Ge_*x*_ (x ≤ 0.625) would not form in some areas resulting in a non-homogeneous dispersion of these defects. Although, N*V* pairs are deemed to bound for all the nearest neighbour environments considered here there are areas that will be likely to form and were there will be more difficult to dissociate. From Fig. 1 we can extract that N*V* pairs will have higher binding energies when there is at least one Si atom at a first nearest neighbour site to the nitrogen atom. For the higher Si content Si_1 − *x*_Ge_*x*_ alloys two or more Si atoms at nearest neighbour positions to the N atom leads to lower binding energies.

**Fig. 2:**
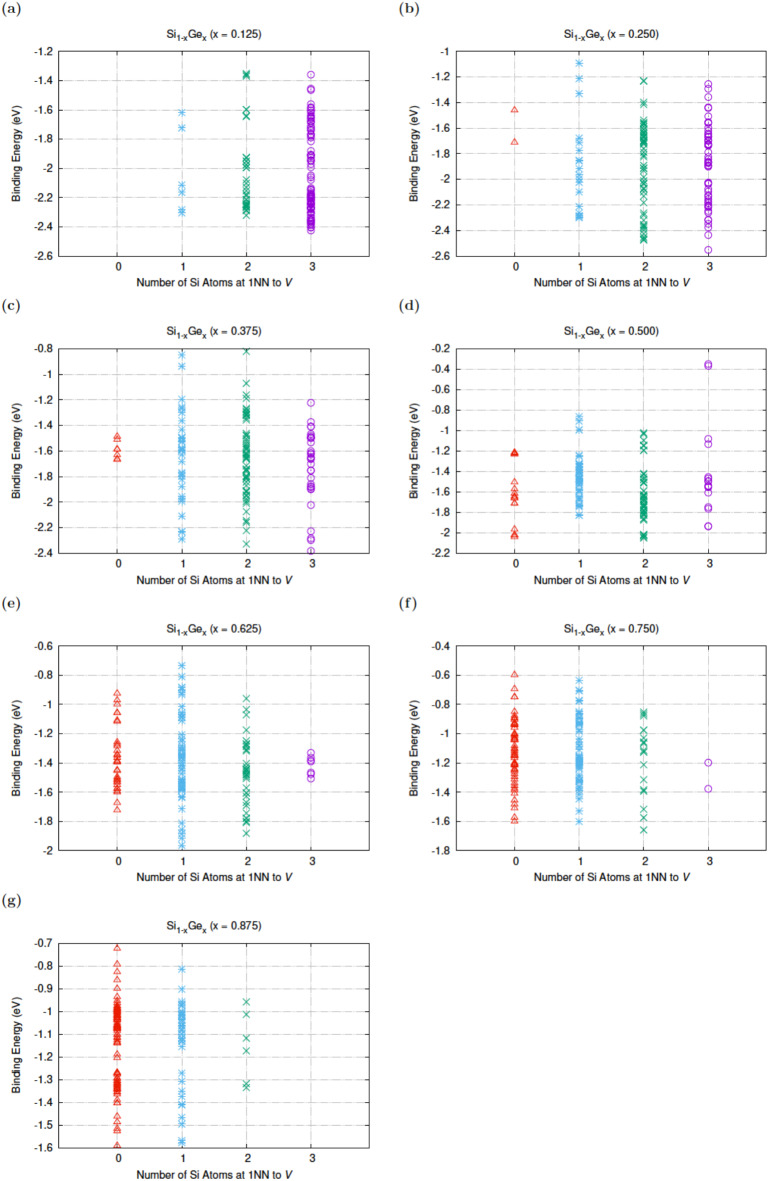
The binding energy of N*V* defects as a function of the number of Si first nearest neighbour atoms to the vacancy in Si_1 − x_Ge_x_ (x = 0.125, 0.25, 0.375, 0.5, 0.625, 0.75, 0.875).

Figure 2 sheds light on the influence of the first nearest neighbour Si or Ge atoms to the lattice vacancy. In previous work on the *E*-centre it was calculated that the most bound configurations irrespective of the Si_1 − *x*_Ge_*x*_ composition will have mostly Ge atoms around the vacancy^40^. This is not the case here as there is significant Si atom presence around the vacant site particularly at high Si content Si_1 − *x*_Ge_*x*_ alloys.

**Fig. 3:**
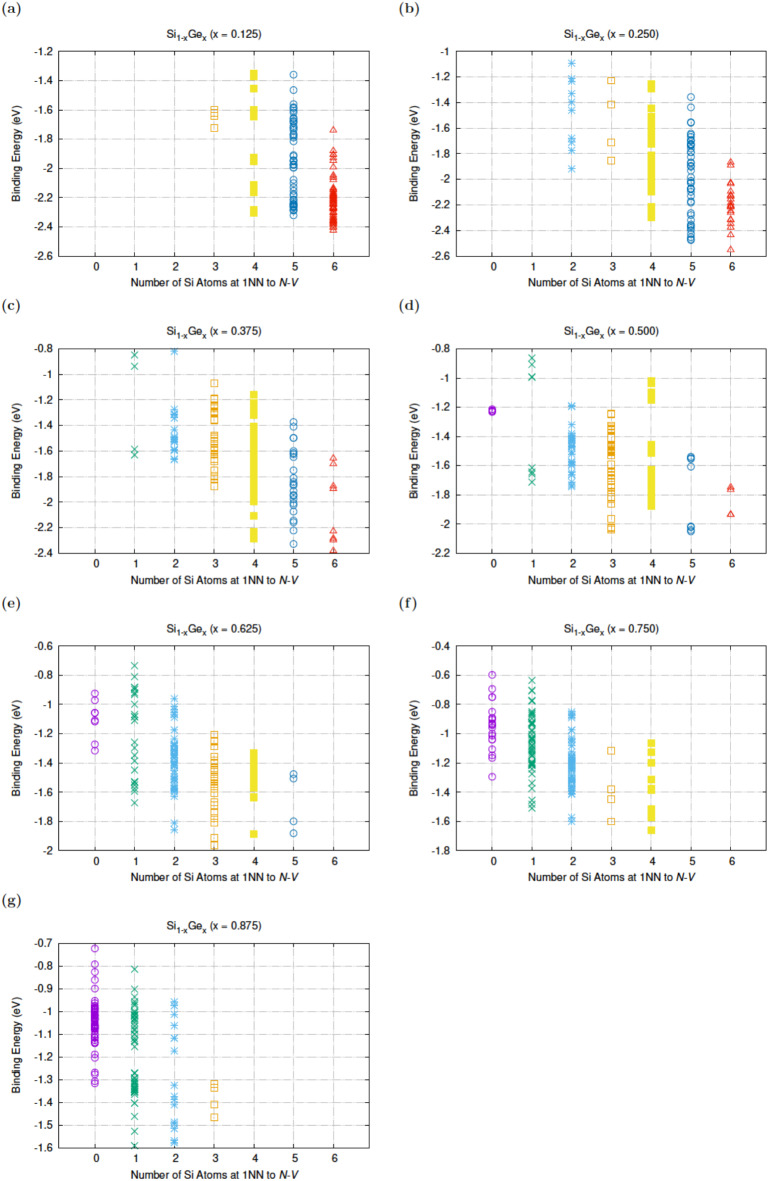
The binding energy of N*V* defects as a function of the total number of Si first nearest neighbour atoms to the vacancy or N in Si_1 − x_Ge_x_ (x = 0.125, 0.25, 0.375, 0.5, 0.625, 0.75, 0.875).

The reason to consider the sum of the nearest neighbours with respect to the nitrogen substitutional and the lattice vacancy is to access whether higher or lower Si content local environments will affect the binding of the N*V* pair. This aims at gaining an understanding of whether the N*V* defects in Si_1 − *x*_Ge_*x*_ prefer to form in Si-rich or Ge-rich regions. Figure 3 adds up the impact of nearest neighbours around the N*V* defect. It can be concluded from Fig. 3 that apart from Si_0.125_Ge_0.875_, N*V* defects preferentially form with 3 or more Si nearest neighbour atoms.

**Fig. 4:**
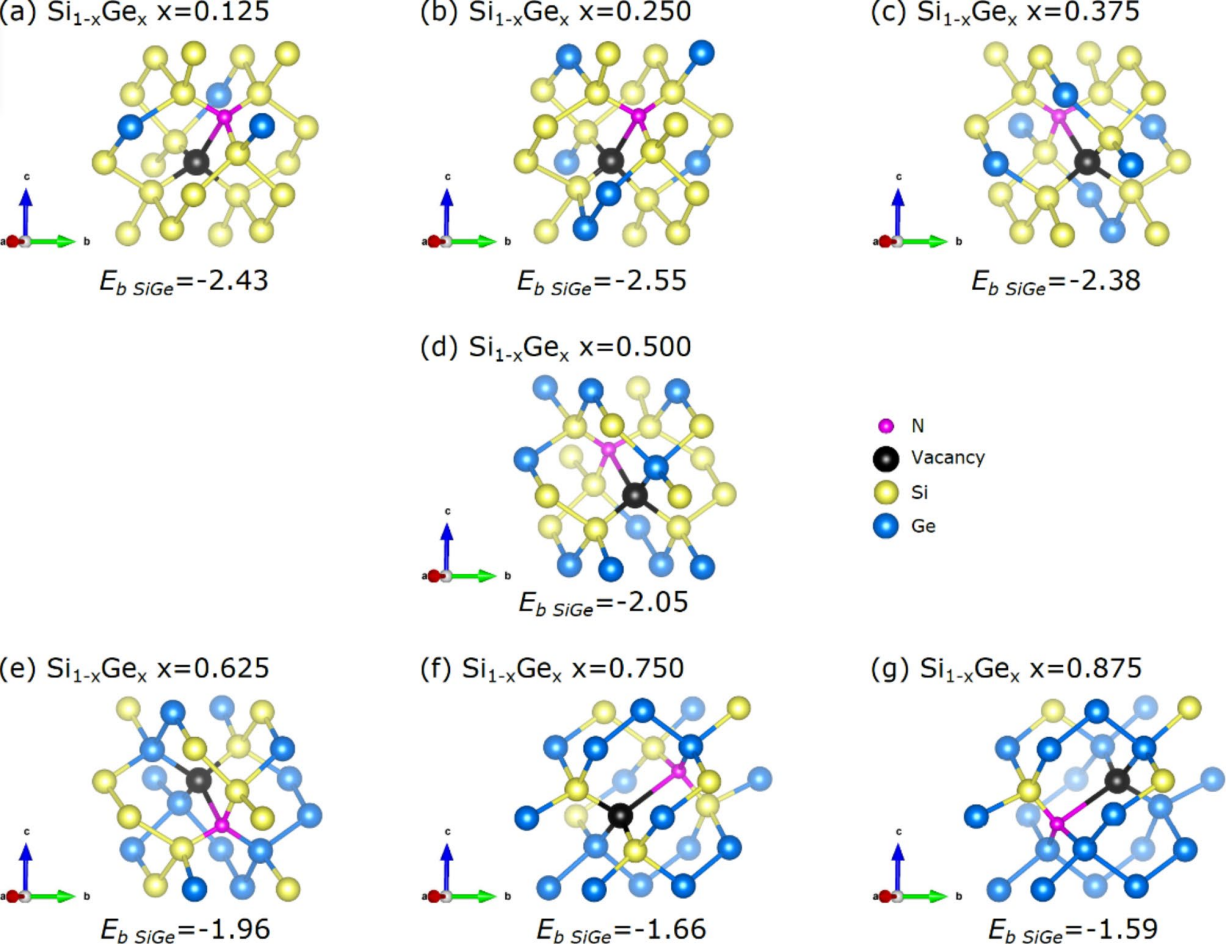
The lowest binding energy N*V* defects and their nearest neighbour atoms in Si_1 − *x*_Ge_*x*_ (x = 0.125, 0.25, 0.375, 0.5, 0.625, 0.75, 0.875). The binding energies (eV) for each composition are given.

The lowest binding energy (i.e. most energetically preferable) N*V* defects and their nearest neighbour atoms for all the Si_1 − *x*_Ge_*x*_ compositions are represented in Fig. 4. The predominantly Si nearest neighbour environments around the N atoms for most compositions is shown (refer to Fig. 4). For the highest Ge content alloy compositions (x = 0.625 and x = 0.75) there are still two Si nearest neighbour atoms to the N. At the highest Ge content considered (x = 0.875) there is only one Si residing at nearest neighbour site to the N atom. The most bound N*V* pairs are significantly more bound as compared to the P*V* pairs considered in a previous study^40^.

**Fig. 5:**
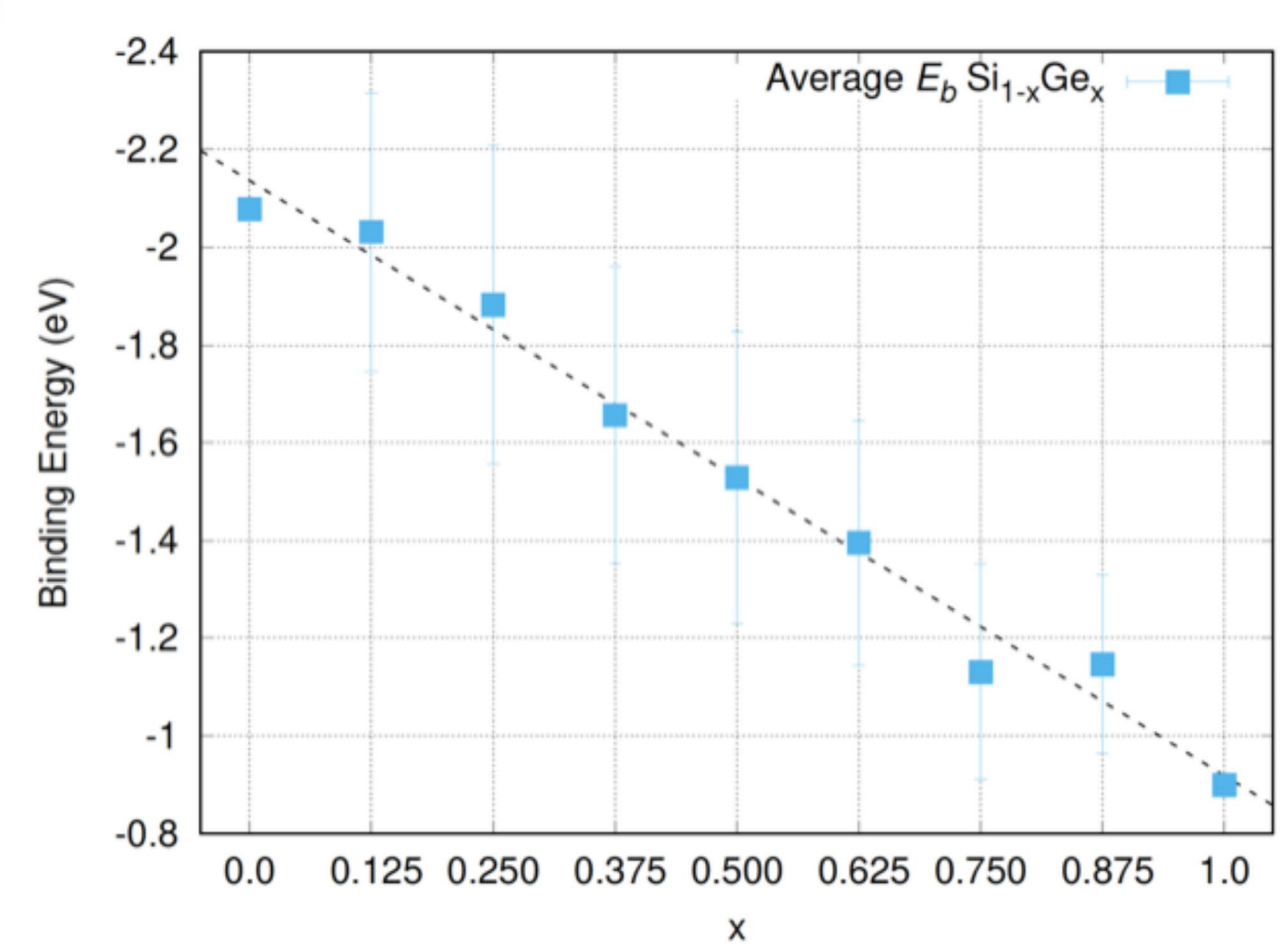
The binding energy dependence of N*V* defects as a function to concentration x in Si_1 − *x*_Ge_*x*_ (x = 0.125, 0.25, 0.375, 0.5, 0.625, 0.75, 0.875).

Figure 5 represents the binding energy dependence of N*V* defects as a function to alloy composition. In previous computational work it was shown that for P*V* defects in Si_1 − *x*_Ge_*x*_ there is deviation from linearity (deviation from Vegard’s law)^5,40^. Here we observe again this deviation from Vegard’s law for the lowest binding energy N*V* defects, however, there is a linear behavior for the average binding energies (refer to Fig. 5).

There is evidence that the dependence of dopant-defect processes in Si_1 − *x*_Ge_*x*_ are non-linear with respect to the alloy composition^4,7,8,40,42,43^. In previous experiments it was determined that the activation enthalpies of diffusion of vacancies, As and Sb do not have a linear dependence as a function of concentration of the Si_1 − *x*_Ge_*x*_ alloy^42,43^. The bowing determined experimentally by Kube et al.^43^. for self-diffusion in Si_1 − *x*_Ge_*x*_ over a wide temperature (963–1543 K) and concentration range (*x* = 0.0, 0.05, 0.25, 0.45 and 0.70) is consistent with the present study. The trends can be comprehended by the c*BΩ* thermodynamic model^44,45^. In this model the macroscopic properties (such as the bulk modulus) can be correlated with microscopic properties (such as diffusivity)^46–49^. In a previous study, Saltas et al.^8^. employed the c*BΩ* thermodynamic to investigate self-diffusion in Si_1 − *x*_Ge_*x*_ and in particular the impact of composition and temperature. In their study Saltas et al.^8^. concluded that the deviations from Vegard’s law for the binding energies of P*V* defects in Si_1 − *x*_Ge_*x*_ were due to the diversification of the bulk properties of Si and Ge. This is also anticipated to be the key reason for the N*V* pairs considered here, which exhibit a similar behaviour to the P*V* defects.

For the *E*-centre there is a clear dependence of the higher Si content alloys leading to stronger defect pairs^40^. Here we observe an analogous trend. What is different though is the local environment of the N*V* pairs as compared to the P*V* pairs. In the latter it was observed that there was a strong Ge atom present at nearest neighbour sites something that is not the case for N*V* pairs. The system gained energy by the Ge atoms surrounding the vacant site in the P*V* pair as the oversized Ge atoms relaxed. As N is a smaller atom compared to P some of the relaxation occurs within the defect pair.

**Fig. 6:**
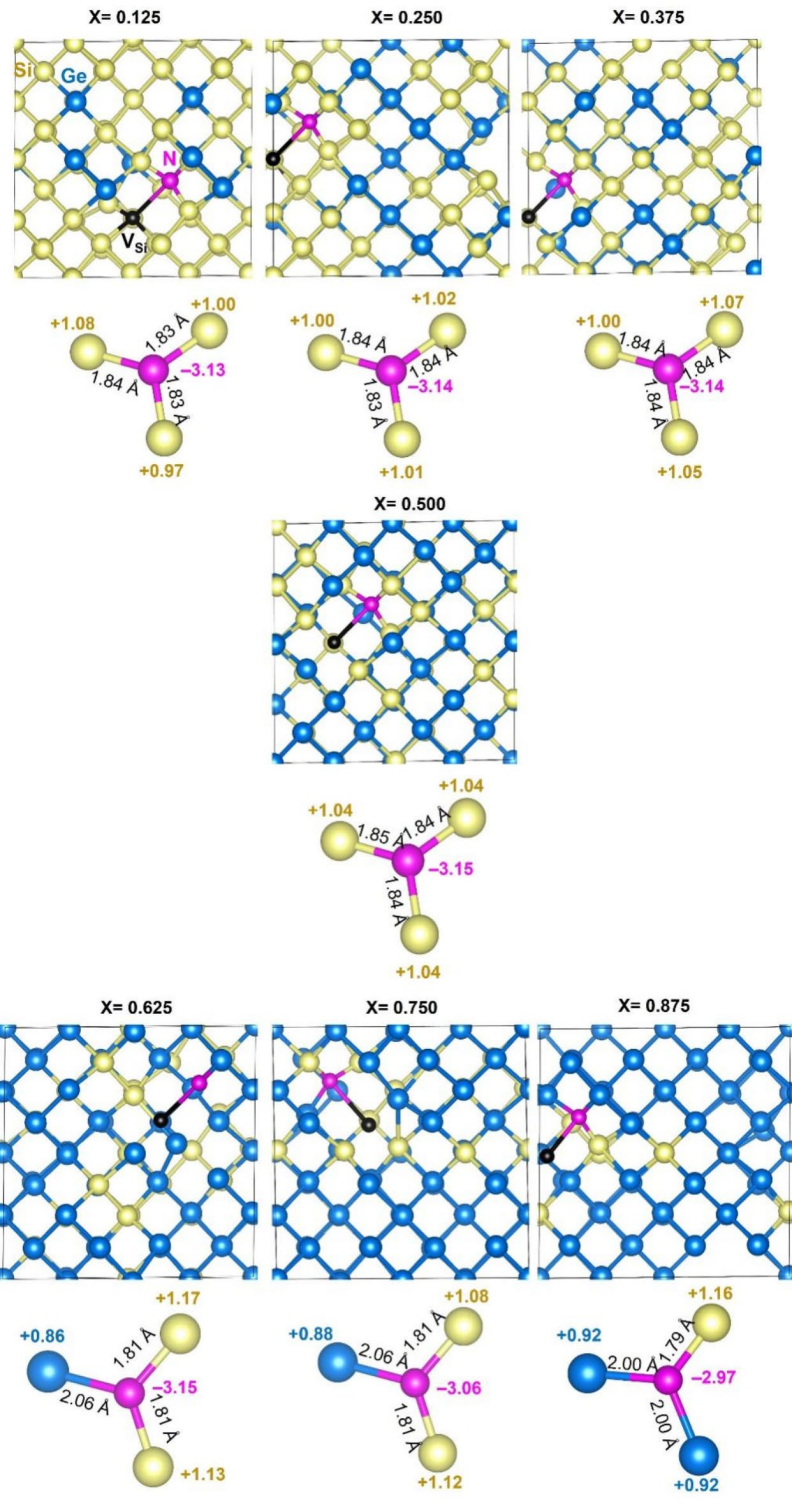
Relaxed structures of seven different nitrogen substitutional-vacancy defect (N*V* pairs) configurations in Si_1 − x_Ge_x_ alloys. Local chemical environments showing bond distances between Si (Ge) and N and Bader charges on N and its nearest neighbour atoms are also shown.

The optimized structures of seven different nitrogen substitutional defect configurations each consisting of a single Si vacancy (*V*_Si_) in Si_1 − x_Ge_x_ alloys were considered to examine the structural parameters, Bader charges and charge accumulation on the nitrogen defects using spin-polarized DFT simulations as implemented in the VASP code^32^. Relaxed configurations of nitrogen substituting a silicon atom with a neighbouring vacancy in Si_1 − x_Ge_x_ are shown in Fig. 6. The substantial interaction between nitrogen and its neighbouring silicon or germanium atoms is clearly observed through the bond distances and Bader charge analysis (see Fig. 6). Nitrogen atoms consistently acquire a negative Bader charge, indicating electron acceptance, while the surrounding Si or Ge atoms exhibit positive Bader charges, reflecting electron donation. The differences in electronegativity between N, Si, and Ge significantly influence the charge distribution within the alloys containing N*V* defects. The positive electronegativity differences (N-Si = 1.14, N-Ge = 1.03)^49^ lead to a substantial negative Bader charge on nitrogen (approximately − 3.00 e), confirming its role as an electron acceptor. This indicates that N forms a stable N^3−^ state. The negative charge on N arises because it receives approximately 1.00 e from each of its three neighbouring Si or Ge atoms. Nitrogen atoms in the alloy form three-coordination bonds with adjacent Si or Ge atoms. This coordination is key to the stability of the N^3−^ state. The Si-N bond distance is consistently around 1.84 Å. The Ge-N bond distance is slightly longer at around 2.05 Å, reflecting the larger atomic radius of Ge compared to Si^50^. This charge transfer significantly impacts the electronic properties of the material and should be considered when analysing or engineering ​Si_1 − x_Ge_x_ alloys for various applications.

**Fig. 7:**
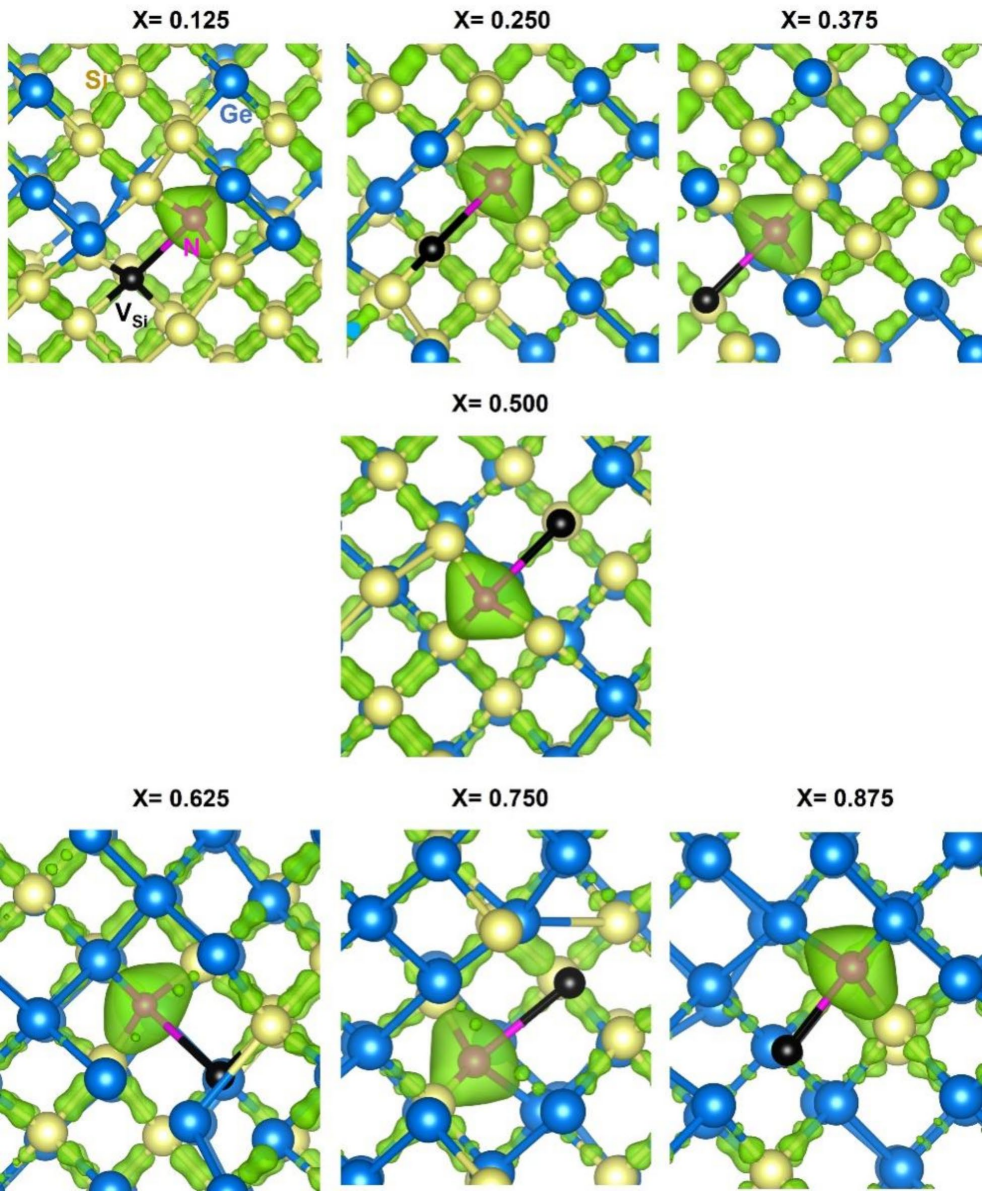
Surfaces of the constant charge density plots associated with the interaction of nitrogen with the nearest neighbour vacancy in each of the seven different configurations in Si_1 − x_Ge_x_ alloys.

Charge density plots (refer to Fig. 7) visually illustrate the electron density concentrated around N atoms, reinforcing the quantitative Bader charge analysis. Each subfigure represents the charge density distribution around a nitrogen atom in all seven configurations, illustrating the high electron density around N and the corresponding bond distances.

A strong interplay between structural distortions and charge distribution in the system involving Si vacancies and Ge incorporation is analyzed (see Table 1). The Si-V_Si_ separation exhibits a strong positive correlation with the Bader charge on Si (Q_Si_), indicating that as the separation increases, Si atoms experience significant charge variations. Additionally, the N-V_Si_ separation shows a moderate negative correlation with Q_Si_, suggesting that vacancy positioning plays a crucial role in charge redistribution. The introduction of germanium leads to notable structural changes, with Ge-V_Si_ separations appearing for x ≥ 0.375 and showing a weak negative correlation with Q_Ge_, implying that shorter Ge separations may result in higher charge accumulation. Overall, the results indicate that vacancies and structural distortions primarily dictate charge behavior, while Ge incorporation influences charge distribution to a lesser extent.

**Table 1:** Bond distances between Si (or Ge) and *V*_Si_, along with bader charges of the nearest neighbouring Si (or Ge) atoms surrounding the *V*_Si_.

x	*N*-V_Si_ (Å)	Si-*V*_Si_ (Å)	Ge*-V*_Si_ (Å)	Q_Si_ (|e|)	Q_Ge_(|e|)
0.125	2.98	2.14, 1.65, 2.14	–	+ 0.05, − 0.02, 0.00	–
0.250	3.00	1.59, 2.10, 2.14	–	0.00, + 0.05, − 0.01	–
0.375	2.52	1.86, 2.78	2.81	− 0.01, + 0.01	+ 0.12
0.500	2.53	2.98, 2.95	1.81	− 0.12, + 1.00	+ 0.15
0.625	3.08	2.15	1.61, 2.95	− 0.04	− 0.05, − 0.17
0.750	2.66	1.71	2.72, 2.97	− 0.05	+ 0.04, + 0.05
0.875	2.33	2.72, 2.93	2.01	− 0.14, + 1.16	+ 1.10

**Fig. 8:**
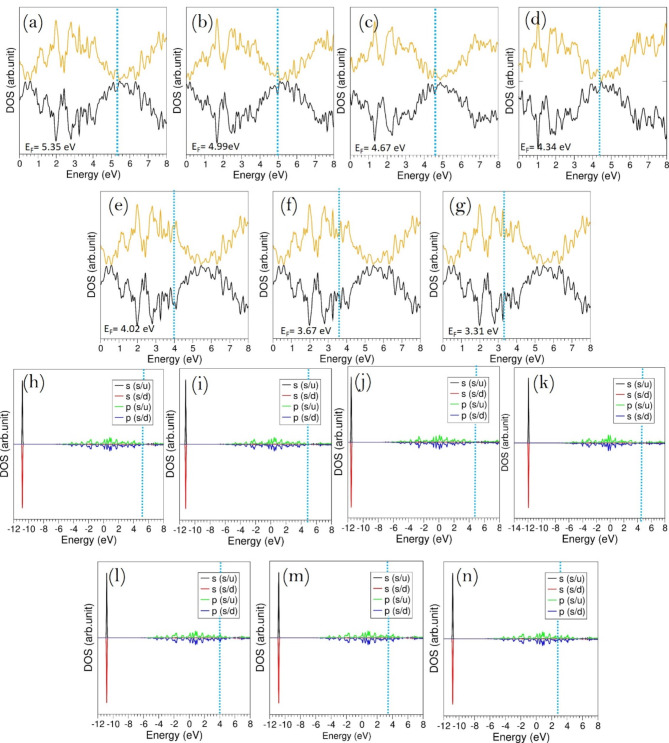
Total DOS plots of NV defects in Si_1 − x_Ge_x_ with (**a**) x = 0.125, (**b**) x = 0.25, (**c**) x = 0.375, (**d**) x = 0.50, (**e**) x = 0.625, (**f**) x = 0.75 and (**g**) x = 0.875. Atomic DOS plots of the N in each corresponding configuration (**h**–**n**) are also shown. The vertical dot lines correspond to the Fermi energy level.

The density of states (DOS) analysis for N*V* defects in Si_1-x_Ge_x_ demonstrates notable changes in the electronic structure as the Ge concentration increases. The total DOS plots (Fig. 8a–g) exhibit a clear downward trend in Fermi energy from 5.35 eV, indicating a shift in electronic properties and a possible reduction in the band gap. This suggests that higher Ge content modifies the defect states and overall electronic distribution, potentially influencing carrier dynamics. The atomic DOS projections (Fig. 8h–n) reveal minimal contributions from nitrogen *p*-orbitals near the Fermi level, while the *s*-states are positioned deeper within the valence band. The interaction of these defect states with electrons introduced by doping suggests notable defect-induced electronic activity, which could impact conductivity and recombination mechanisms. Overall, these findings indicate that adjusting the Si-Ge composition enables precise control over electronic properties, making it valuable for semiconductor applications, especially in defect-engineered quantum devices and optoelectronics.

All defect configurations displayed zero spins, confirming their non-magnetic nature. This indicates that the incorporation of NV defects in Si_1-x_Ge_x_ does not generate significant magnetic moments, suggesting that these defects primarily impact the electronic properties rather than contributing to magnetism. The lack of spin polarization further reinforces the notion that charge carrier dynamics and defect-induced electronic states govern the behaviour of the system, making these materials more applicable to electronic and optoelectronic technologies rather than spintronic devices.

## Conclusion

In the present investigation, we have used extensive DFT calculations to consider vacancy-nitrogen defects in Si_1 − x_Ge_x_. For all the concentrations considered N*V* pairs are bound. Importantly they are significantly more bound than the P*V* defects considered in previous work. The N*V* is different to the P*V* pair in Si_1 − *x*_Ge_*x*_ and this is also reflected in the nearest neighbour environments of the lowest binding energy defects. In N*V* pairs there are less Ge atoms surrounding the vacant site.

## Data Availability

Data availabilityThe datasets used and/or analysed during the current study available from the corresponding author on reasonable request.
